# Rilonacept (IL-1 TRAP) for treatment of colchicine resistant familial mediterranean fever (FMF): a randomized, multicenter double-blinded, alternating treatment trial

**DOI:** 10.1186/1546-0096-9-S1-O38

**Published:** 2011-09-14

**Authors:** PJ Hashkes, SJ Spalding, EH Giannini, B Huang, G Park, KS Barron, MH Weisman, N Pashinian, AO Reiff, J Samuels, D Wright, DL Kastner, DJ Lovell

**Affiliations:** 1 Cleveland Clinic Foundation, OH, USA; 2 Cincinnati Children's Hospital Medical Center, OH, USA; 3 National Institutes of Health, MD, USA; 4 Cedar-Sinai Medical Center, CA, USA; 5 Children’s Hospital of Los Angeles, CA, USA; 6 NYU Langone Hospital for Joint Diseases, NY, USA; 7 Children’s Hospital Central California, CA, USA; 8 Shaare Zedek Medical Center, Jerusalem, Israel

## Background

There is no current treatment alternative for patients with FMF whose disease is resistant to, or do not tolerate colchicine. Since pyrin has an important role in IL-1β regulation we hypothesize that IL-1 inhibition will decrease the number of FMF attacks in these patients.

## Aim

To determine if rilonacept, a fusion protein that binds and neutralizes IL-1, decreases the number of FMF attacks compared to placebo.

## Methods

Subjects were FMF patients ≥4 years of age recruited at 6 U.S. sites, who had at least 1 FMF attack per month despite receiving adequate doses of, or who were intolerant of colchicine. Subjects received two 3-month courses of rilonacept (Arm A) at 2.2 mg/kg (max 160 mg) by weekly SC injection and two 3-month courses of placebo (Arm B). Patients were randomized to 1 of 4 treatment sequences (ABAB, BABA, ABBA, BAAB). Escape visits were allowed to permit switching arms (blinding was maintained) for patients with at least 2 attacks within a course. The primary outcome was the difference of FMF attacks between rilonacept and placebo courses with responders defined as subjects with a >40% difference. Results were analyzed by paired t-and signed rank tests.

## Results

Fourteen subjects were randomized, 8 males and 6 females, mean (±SD) age 24.4±11.8 years (range 4.5-47.3; 4 patients <18 years), disease duration 17.5±12.6 yrs, with a baseline of 3.1±2.0 attacks per month. Eleven completed the full study and 3 dropped out (1 due to lack of efficacy, 1 due to distance from study site and 1 lost to follow-up). Among 12 patients who completed at least 2 treatment courses the mean number of attacks per month on rilonacept was 1.0±1.2 vs. 1.8±0.9 on placebo (P=0.021 by paired t-test and 0.027 by signed rank test). There were 8 responders; all 4 non-responders were adults. There were 2 respiratory infection SAEs, 1 on rilonacept and 1 on placebo. Injections site reactions were significantly more frequent with rilonacept but no differences were seen in other adverse events, including infections.

## Conclusions

Rilonacept significantly reduced the number of FMF attacks and had an acceptable safety profile. IL-1 inhibition is a treatment option for most (especially children) colchicine resistant FMF patients.

